# Trends in the health status of Ukrainian refugees in Norway according to month of arrival during 2022

**DOI:** 10.1186/s12889-024-20660-0

**Published:** 2024-11-12

**Authors:** Angela S. Labberton, Larisa Ozeryansky, Ylva Helland, Thea Steen Skogheim, Tonya Moen Hansen

**Affiliations:** 1 Division for Health Services, Norwegian Institute of Public Health, Postboks 222, Skøyen, Oslo, 0213 Norway; 2 The Graduate School, The University of Washington, Seattle, USA; 3 The Norwegian Directorate of Health, Oslo, Norway

**Keywords:** Refugees, Health needs assessments, Mental health, Oral health, Chronic disease

## Abstract

**Background:**

More than 35 000 refugees from Ukraine applied for temporary collective protection in Norway during 2022. Previous studies have shown that the refugees have poor health in several domains, and crude reports have suggested that those fleeing Ukraine at later stages have even poorer health. However, more systematic knowledge is lacking. This study aimed to analyse trends in self-reported health in a sample of adult refugees from Ukraine, by month of arrival to Norway during 2022.

**Methods:**

Data were collected via an online, digital questionnaire, in a cross-sectional study design between 28.10.22–31.01.23. Recruitment was via multiple physical and social media contact points, including asylum reception centres, municipalities, non-profit organisations, and Facebook groups for refugees in Norway. The survey included the following self-reported health outcomes: overall health, oral health, presence of long-term illnesses or disabilities, and a short version of the Hopkins Symptom Checklist (HSCL-5).

**Results:**

Among the 727 respondents, 82% were female, 65% were aged 30–49 years, 69% had higher education and 53% were responsible for children in Norway. There were 383 respondents who arrived between February-April (T1), 200 between May-August (T2) and 144 between September-December (T3). Compared to T1, respondents who arrived in the two later time periods were more often male, had younger age distributions, and were less likely to have completed higher education. The proportions of respondents reporting poor/very poor overall health, presence of long-term illnesses and long-term disabilities were highest in T3. Oral health and HSCL-5 showed the opposite trend, with lower proportions reporting poorer health among respondents in both later periods. Respondents in T3 were still more likely to report poor/very poor health and long-term illnesses after adjusting for sex, age group and education (adjusted odds ratio, aOR: 2.71 [95%CI 1.51–4.89]) and 1.74 [1.14–2.65], respectively).

**Conclusions:**

Respondents who arrived later in 2022 generally reported poorer long-term health, but less psychological distress than those who arrived earlier in the year. These findings may help inform the planning of health services for refugees from Ukraine, especially in areas receiving large numbers of refugees.

## Background

The full-scale invasion of Ukraine in February 2022 has resulted in one of the largest displacement crises in the world today [1]. As with other European countries, Norway offered temporary collective protection (TCP) to refugees from Ukraine [2]. During 2022, over 35 000 people from Ukraine applied for TCP in Norway [3]. This group represents the largest-ever single wave of refugees to have come into Norway in one year. In comparison, there were in total 2 305 asylum applications in 2019 – representing a ‘normal’ year – and around 10 500 asylum seekers from Syria in 2015 [4, 5].

In this article, we refer to persons fleeing Ukraine due to the war as ‘refugees’ in the common use of the term and for simplicity. In Norway, collective protection is regulated by the Immigration Act, Sect. 34, and collective protection is granted as a residence permit for one year at a time [2]. There is a growing body of literature on legal status, health care access, and associated health and health outcomes among migrants and refugees [6–9]. Extended time in uncertainty and being subject to temporary living conditions over time can impact health, especially mental health [7–9]. Beneficiaries of TCP experience expedited processing of their application. Like all asylum seekers in Norway, they have the same health care rights as the rest of the population from the time they submit their application. These rights include access to primary and specialist healthcare at the same cost as for locals. Thus, relative to other asylum seekers and resettled refugees, Ukrainians have fewer legal hindrances and a shorter journey to Norway. However, they may still face barriers when attempting to access health care, such as language, lack of resources, and unfamiliarity with the health care system.

There has been a lack of systematic information about the health and healthcare needs of refugees from Ukraine, which may differ significantly from that of other refugees. The refugees leaving Ukraine have mainly been women, children and elderly, and therefore differ demographically from refugees previously coming to Norway, the majority being adult men [10]. It is also uncertain how transferable knowledge about the health status of the general Ukrainian population is to the persons fleeing Ukraine due to the full-scale invasion.

The Norwegian Institute of Public Health (NIPH) published a report in May 2023 with findings that refugees from Ukraine arriving during 2022 reported overall poorer health, compared to a weighted sample of the Norwegian population [11]. Compared to the Norwegian sample, a smaller proportion of the refugees rated their own health as good or very good (48% vs. 73%), a larger proportion reported having long-term illnesses or health problems (58% vs. 44%), and having psychological distress according to the 5-item version of the Hopkins Symptom Checklist (32% vs. 11%). Furthermore, only 31% of the refugees rated their oral health as good or very good, compared to 74% in the Norwegian sample [11, 12]. The survey findings indicate a great need for healthcare services, including continuity of treatment, dental care and mental health support. These findings are similar to studies on Ukrainian refugee health in other European countries, specifically the findings that a variety of health indicators were poor and that more health-based support was needed for the refugees as compared to the local population [13–16].

The survey also assessed the information they had received about health services in Norway, and whether they had received the health care they felt they had needed. Findings show that the refugees who had most recently arrived were less likely to have received understandable information about health services in Norway, less likely to know how to contact the health services, and less likely to have received the health care they felt they needed, as compared to those refugees that had been longer in Norway [11, 17]. There appears, therefore, to be a time trend regarding the refugees’ self-reported knowledge about health services in Norway and whether they had received the health care they felt they needed.

Health status and healthcare needs among refugees are not static and may depend on the current situation in their homeland and the conditions of their flight, thus showing a temporal trend. Mental health challenges in refugee populations have been well-studied; a meta-analysis of prevalence rates for post-traumatic stress disorder (PTSD) and depression amongst refugee adults found that 30% experienced PTSD and more than 30% experienced major depression [18]. What seems to be less-studied, however, is how psychological distress can change even after a refugee has left their war-time context and spent time in a resettlement process.

The health status of the refugees leaving Ukraine in the early stages of the invasion may differ from those leaving later. Those with better health may have been able to flee more easily and earlier whilst those with greater health issues may attempt to postpone as long as possible. Alternatively, those who arrived later may have poorer health after having been exposed to the consequences and context of war or having lived in temporary living conditions for longer. There is a lack of knowledge on temporal trends in the health status of Ukrainian refugees, and this paper aims to explore this among the refugees in Norway.

### Aim

To investigate whether health status differs between refugees who arrived earlier and those who arrived later to Norway, accounting for changes in demographic factors.

## Methods

### Study design and data collection

The study design was cross-sectional with three comparative time points to investigate temporal trends in outcomes. Data collected by NIPH were used in the current study [11]. Data were collected via an online, digital questionnaire in a cross-sectional study design between 28th October 2022–31st January 2023. Printed and digital posters and brochures with information about the survey and links to the questionnaire were distributed to multiple physical and social media contact points for refugees, including the National Arrivals Centre and ordinary asylum reception centres, municipalities, non-profit organisations, and Facebook groups for refugees in Norway. Study information and the questionnaire were available in Ukrainian, Russian, English and Norwegian. All participants were required to give informed consent before accessing the questionnaire. A copy of the questionnaire in English is available in Appendix A of the NIPH report [11].

Physical contact points were contacted via email with information about the study and an invitation to participate as a point of contact for recruitment, including digital versions of the poster and flyer in Ukrainian and Russian to be hung up or distributed to refugees from Ukraine. Printed versions of the poster and flyers were also offered to be sent out to the physical contact points by post. If no reply to the initial email contact was received, an email reminder was sent after approximately three weeks. Information and a link to the survey, were also posted several times during the recruitment period to the Facebook groups, and NIPH’s migration health Facebook page and mailing list.

### Survey questionnaire

The questionnaire and information material were developed in close collaboration with a native Ukrainian living in Norway and qualified translator in Ukrainian and Russian, and colleagues experienced in survey design and refugee health. The Ukrainian and Russian translations were performed by the qualified translator and independently checked by native speakers working at the NIPH. The English translation was performed by a native speaker with experience in translations, and independently checked by a second native speaker at NIPH. Any disagreements were discussed until a final translation was agreed upon.

The questionnaire collected sociodemographic information: sex, age group (10-year categories), highest completed education and whether they were responsible for child(ren) in Norway. Several health-related survey items were taken from previous population surveys (the Norwegian County Public Health Surveys, NCPHS). Items used in the present study were overall self-rated health and dental health via a 5-point Likert scale: ‘Very good’, ‘Good’, ‘Fair’, ‘Poor’, or ‘Very poor’. The presence of any long-term illnesses or health problems (‘Yes’ or ‘No’), or long-term disabilities or problems due to injury (‘Yes’ or ‘No’). Long-term was specified as conditions that had lasted, or were expected to last, at least six months, and including problems that come and go. A short version of the Hopkins Symptom Checklist (HSCL-5) measuring symptoms of depression and anxiety [19] was also included. The HSCL-5 comprises five questions about how much the respondent has been bothered by the following symptoms during the last week: nervousness or shakiness inside, feeling fearful, feeling hopeless about the future, feeling blue or sad, and worrying too much about things [20]. Each question has four answer options: ‘Not at all’ (1 point), ‘A little’ (2 points), ‘Quite a bit’ (3 points), and ‘Extremely’ (4 points).

Self-reported need for health services due to long-term health problems (number of visits annually) was used to assess internal consistency in the current study.

### Data management

The project was approved by NIPH’s Data Protection Officer after reviewing the project’s data protection impact assessment (DPIA). No directly identifying information was collected. Data collection and data storage was carried out using Nettskjema, an online tool for creating, storing, and managing surveys and data collections, and Services for Sensitive Data (Tjenester for Sensitive Data, TSD), both developed by the University of Oslo. Data were stored and analysed on NIPH’s secure server.

### Study outcomes

The outcomes of interest in the current study were indicators of poor health, defined as the following dichotomous outcomes: ‘poor’ or ‘very poor’ overall self-rated health, ‘poor’ or ‘very poor’ oral health, mean HSCL-5 score above 2, presence of long-term illnesses or health problems, and presence of disabilities or problems due to injury. These outcomes were analysed according to month of arrival in 2022 of the respondents: February to April (T1), May to August (T2), or September to December (T3).

### Statistical analyses

Responses with missing month of arrival or missing values on the outcomes of interest were excluded from the analyses. For HSCL-5, the mean score was calculated across the five items giving a value between 1 and 4. We followed NCPHS [21] and Strand et al. [20] in using a cut-off mean score of 2, above which indicates psychological distress. Respondents with missing values on more than one item were excluded from these analyses [20].

Categorical variables are presented as frequency and percentage. Differences in proportions were analysed using two-sided proportional tests with Pearson’s chi-squared test statistic. We estimated adjusted odds ratios (aORs) for each outcome by month of arrival using binomial logistic regression, adjusting for sex, age group, and education (dichotomised as completed higher education or not).

Finally, internal consistency was assessed by regressing self-reported need for health services due to long-term health problems of more than 5 visits per year against reported poor/very poor overall health and oral health.

A 5% significance level was applied, using two-sided tests. Statistical analyses were performed using R Studio version 2023.12.1 + 402 [22].

## Results

### Study participants

In total 739 survey responses were received. Responses were excluded from analysis if they had: missing arrival month (*n* = 4), missing values on the outcomes of interest (*n* = 8), and for calculation of mean HSCL-5 score: missing values on any of the items on the HSCL-5 (*n* = 1). A total of 727 survey responses were therefore included in analyses (726 for mean HSCL-5). Internal consistency was good, as shown by reported poor/very poor health and oral health being highly predictive of reporting a need for health services five or more times per year.

Participants were predominantly female (82%), aged between 30 and 49 years (65%), and with higher education (69%) (Table 1). Just over half of the respondents were responsible for children in Norway (53%).

**Table 1:** Description of the participants, by month of arrival in 2022

	Whole sample	Arrived February - April (T1)	Arrived May - Aug (T2)	Arrived September - December (T3)
*n*	727	383	200	144
Female, *n* (%)	599 (82.4)	346 (90.3)	151 (75.5) ***	102 (70.8) ***
Age in years, *n* (%)				
18–29	130 (17.9)	65 (17.0)	32 (16.0)	33 (22.9) ***
30–39	264 (36.3)	131 (34.2)	74 (37.0)	59 (41.0)
40–49	210 (28.9)	119 (31.1)	58 (29.0) **	33 (22.9) ***
≥ 50	122 (16.8)	68 (17.8)	36 (18.0) *	18 (12.5) *
Completed higher education, *n* (%)	499 (68.6)	277 (72.3)	131 (65.5) ***	91 (63.2) ***
Born in Ukraine, *n* (%)	665 (91.5)	354 (92.4)	181 (90.5) ***	130 (90.3) ***
Responsible for child(ren) in Norway, *n* (%)	384 (52.8)	217 (56.7)	101 (50.5) **	66 (45.8) ***

Note: *** *p* < 0.001, ** *p* < 0.01, * *p* < 0.05. Proportions for respondents arriving in T2 and T3 compared to respondents arriving in T1 using two-sided proportional test with Pearson’s chi-squared test statistic

Over half of the respondents arrived between February and April 2022 (T1, 53%). The T1 group had a higher proportion of females (90%), with higher education (72%) and with children (57%), compared to those who arrived later in the year (Table 1). Notably, there were fewer respondents aged 50 years or over among respondents who arrived later, with a statistically significant lower proportion among those who arrived in T3, September to December (12.5% vs. 17.8% in T1, *p* < 0.05)).

### Health outcomes by month of arrival

**Fig. 1:**
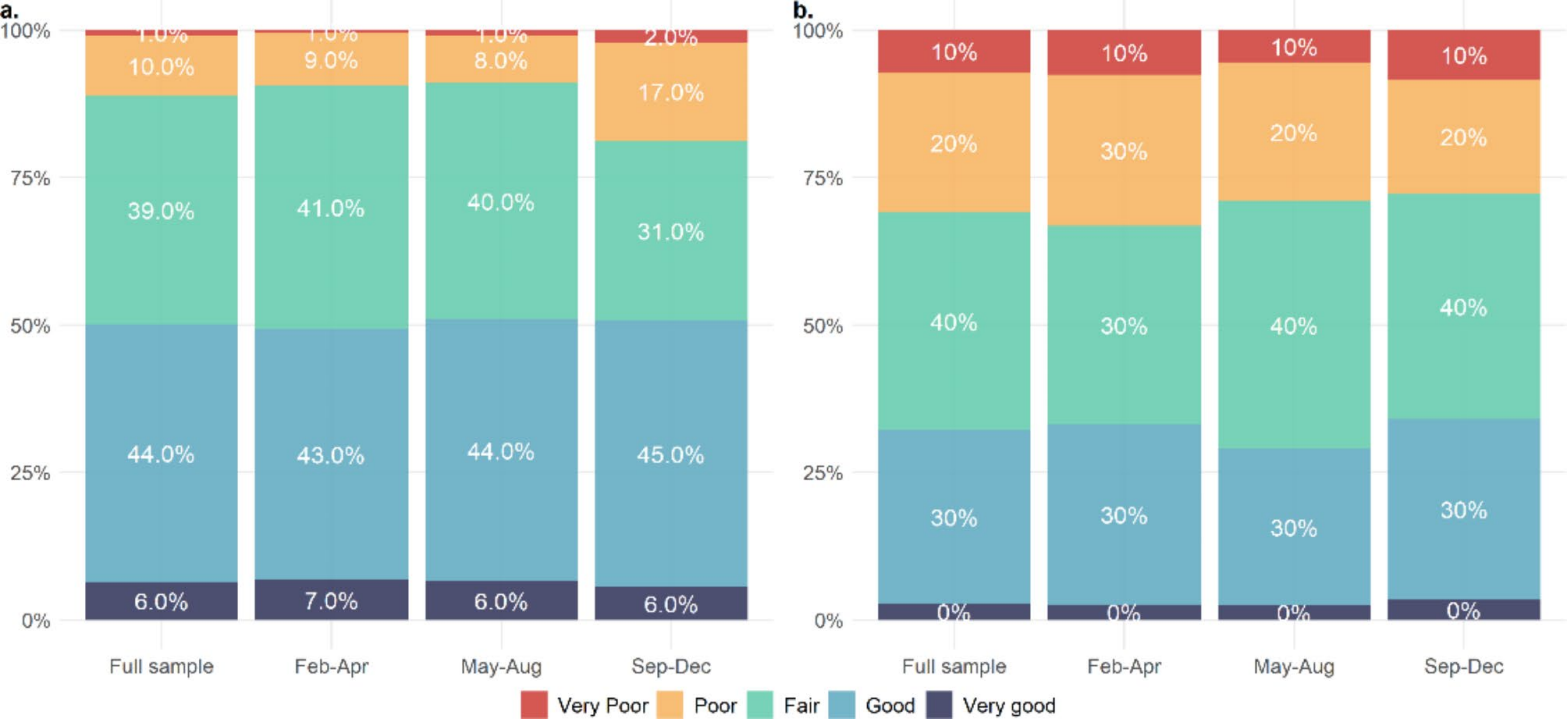
Distribution of answer categories by arrival month: **a**. self-rated overall health, **b**. self-rated oral health. Feb-Apr = Arrived February til April 2022 (T1); May-Aug = Arrived May til August 2022 (T2); Sep-Dec = Arrived September til December 2022 (T3)

Table 2 shows proportions reporting poor health outcomes according to arrival month in 2022, and Fig. 1 shows differences for all categories for outcomes for (a) self-rated overall health and (b) oral health.

**Table 2:** Number of and proportion of respondents, by month of arrival, for primary outcome measures

	Whole sample	Arrived February - April (T1)	Arrived May - August (T2)	Arrived September -December (T3)
*n*	727	383	200	144
Overall health poor or very poor, *n* (%)	81 (11.1)	36 (9.4)	18 (9.0)	27 (18.8) **
Has a long-term illness or health problem, *n* (%)	416 (57.2)	210 (54.8)	115 (57.5) ***	91 (63.2) ***
Has a long-term disability or problem due to injury, *n* (%)	119 (16.4)	55 (14.4)	36 (18.0) *	28 (19.4) ***
Dental health poor or very poor, *n* (%)	225 (30.9)	127 (33.2)	58 (29.0) *	40 (27.8) **
Mean HSCL-5 score > 2, *n* (%)	245 (33.7)	141 (36.8)	63 (31.5) *	41 (28.5) **

Note: *** *p* < 0.001, ** *p* < 0.01, * *p* < 0.05. Proportions for respondents arriving in T2 and T3 compared to respondents arriving in T1 using two-sided proportional test with Pearson’s chi-squared test statistic

The proportion of respondents who reported that their overall health was poor or very poor was higher among the latest arrivals (T3), compared to those who arrived in T1: 18.8% vs. 9.4%, *p* < 0.001. There was a statistically significant trend for long-term illnesses or health problems, and for long-term disability or problems due to injury, where both later groups (T2 and T3) reported more long-term illness or disability compared to those who arrived in T1. There was 8.1%-point difference between T1 and T3 for long-term illnesses, and 5.0%-point difference for disabilities.

Oral health showed an opposite trend, with lower proportions reporting poor or very poor oral health among respondents in both T2 and T3 compared to T1. There was a 5.4%-point difference between T1 and T3.

The proportion reporting psychological distress, as measured by a mean HSCL-5 score of greater than 2, was higher among those who arrived in T1, compared to those arriving in T2 and T3: 36.8% vs. 31.5% and 28.5% respectively.

### Multivariable analyses

The results from multivariable logistic regressions for each outcome by month of arrival, adjusted for sex, age group, and education, are shown in Table 3. Following these adjustments, respondents who arrived in T3 were still more likely to report poor or very poor overall health (aOR: 2.71 [95% confidence interval, CI: 1.51–4.89]), and long-term illnesses or health problems (aOR 1.74 [95% CI 1.14–2.65]), compared to those who arrived in T1. Differences in proportions reporting disabilities or injury, poor or very poor oral health, and psychological distress were fully attenuated after adjusting for sex, age group and education.

**Table 3:** Results from multivariate binomial regression models estimating main health outcomes by month of arrival

	Overall health poor or very poor	Has long-term illness or health problem	Has long-term disability or problem due to injury	Dental health poor or very poor	Mean HSCL-5 score above 2
*n*	720	720	720	720	719
***Month of arrival in Norway***					
February – April (T1)	Ref.	Ref.	Ref.	Ref.	Ref.
May – August (T2)	0.972 (0.521, 1.814)	1.197 (0.832, 1.722)	1.207 (0.751, 1.942)	0.766 (0.518, 1.132)	0.859 (0.592, 1.248)
September – December (T3)	**2.713** **(1.506**,** 4.890)**	**1.741** **(1.143**,** 2.653)**	1.333 (0.786, 2.260)	0.741 (0.475, 1.157)	0.735 (0.477, 1.132
***Adjusted for***					
Sex: (Ref: Female)					
Male	0.792 (0.407, 1.541)	**0.604** **(0.398**,** 0.916)**	1.516 (0.920, 2.499)	1.106 (0.712, 1.719)	**0.476** **(0.295**,** 0.769)**
Age group: (Ref.: 18–29 years)					
30–39 years	0.591 (0.275, 1.270)	1.395 (0.888, 2.190)	1.785 (0.909, 3.507)	1.005 (0.621, 1.628)	1.054 (0.673, 1.650)
40–49 years	0.795 (0.369, 1.713)	**1.860** **(1.209**,** 2.861)**	1.849 (0.926, 3.693)	1.067 (0.650, 1.752)	0.738 (0.459, 1.213)
≥50 years	**3.531** **(1.742**,** 7.156)**	**4.428** **(2.533**,** 7.741)**	**3.208** **(1.574**,** 6.536)**	**2.258** **(1.326**,** 3.844)**	0.902 (0.532, 1.528)
Education: (Ref.: Not completed higher education)					
Completed higher education	**0.526** **(0.319**,** 0.867)**	0.856 (0.605, 1.211)	**0.622** **(0.407**,** 0.953)**	**0.557** **(0.392**,** 0.790)**	0.784 (0.552, 1.113)

Note: Ref. = reference category. Results shown as adjusted odds ratio (95% confidence interval), adjusted for month of arrival in addition to sex, age group, and education. Adjusted odds ratios with 95% confidence intervals not crossing 1 are highlighted in bold

## Discussion

This study shows that respondents who arrived in Norway at later stages of 2022 generally reported poorer health, and more frequently long-term illnesses and disabilities than those who arrived during the first period. However, the respondents who arrived later also reported less psychological distress during the last week, and slightly better oral health. There were fewer older respondents in the later time periods, and a higher proportion of men. Differences in demographics over time appeared to drive several of the observed differences in health status.

Studies on temporal trends in the health status of the refugees from Ukraine are lacking. Colleagues from Ireland observed temporal changes in age and sex distributions and prevalence of some chronic diseases among recipients of TCP who had undergone a health needs assessment [23]. Similarly, more primary care physicians in Poland reported having contact with Ukrainian refugees due to chronic disease during February 2023 as compared to April 2022, with an increase from 49 to 70% for cardiovascular conditions [24]. Health services in Norwegian municipalities that have received Ukrainian refugees have reported an apparent increase in persons with complex health needs among those who have arrived more recently. Our data from 2022 show a similar trend, however systematic trends in health status beyond this time are uncertain.

Most survey respondents were female with higher education, particularly among those who arrived during T1. There were fewer respondents in the older age groups, especially among the later arrivals, nevertheless, those arriving later reported poorer overall health and more long-term illness and disability. Based on demographics alone, it can be expected that there would be a relatively high use of health services, since females generally have a higher use than males, particularly during the fertile years [25]. The changes in demographics we observe with higher proportions of men and fewer with children during the later periods, reflect the general trends seen in official registrations [26].

Our survey was based on existing public health surveys in Norway and most outcomes pertain to long-term health problems. The HSCL-5 is the only survey item that captures respondents’ acute health status by asking specifically about symptoms during the last week. The refugees from Ukraine who arrived earlier (T1) reported more psychological distress at the time of the cross-sectional survey than those that arrived later (T2 and T3). Multivariable regressions indicate that being female was independently associated with psychological distress. This association has been shown in several other studies among asylum seekers and resettled refugees globally and in a Scandinavian context as well [9, 27, 28]. Our survey is not able to determine whether the longer time spent in Norway also might play a role. Previous findings on refugee mental health have theorised that the mental burden of trauma can worsen over time, that post-resettlement and transitional stressors can negatively impact mental health [29–31], and that exposure to armed conflict is associated with increased prevalence of mental health disorders, depression, PTSD, and anxiety among refugees [32]. These findings speak to the unique context of the crisis in Ukraine, which required most men to stay behind and fight - separating refugees from family and leading to many female refugees managing childcare responsibilities alone and far from the cultural context of home [33]. Finally, the expectations of eventual return to Ukraine, inherent in the allowance for “temporary” protection, may contribute to refugees feeling that they are in a “liminal state”, which has been associated with feelings of regret and guilt due to family separation, inability to care for family needs, the disruption in the lives of children, and a general lack of control over one’s life circumstances [33].

Oral health was also included in the survey. Overall, 31% of respondents reported that their oral health was poor or very poor, with slightly lower proportions over the three time periods. Adjustments for differences in sociodemographic factors attenuated the temporal trend, with older age and not having higher education being independent predictors of poor oral health. Poor oral health reflects findings from another European survey among refugees [16], and Ukraine has a high prevalence of caries, especially in areas with low fluoride content in the drinking water and radiation-contaminated areas [34]. Furthermore, there is evidence of disparities in access and quality of oral health care in Ukraine, particularly between rural and urban areas [34, 35].

The trend we observe regarding generally poorer long-term health among later arrivals may be partially explained by the healthy immigrant theory [36, 37] which stems from the assumption that people with better health (and resources) can more easily migrate. It is feasible that this also applies to refugees, and that those who arrived during the first influx were generally healthier and more resourceful, compared to those who came later [38]. Ukrainians have been granted TCP in European countries, removing many legal obstacles and requirements usually associated with migration. Still, there are several reasons why those with poorer health may not have left early on, including a lack of resources ‒ since socioeconomic status is closely related to health ‒ or not wanting to interrupt ongoing health care in Ukraine.

Another explanation could be that those arriving later have poorer health due to being exposed to war and disruptions in healthcare for longer. Our survey only recorded the month of arrival in Norway, not the time of departure from Ukraine, so we do not know if respondents spent time in another country before arrival in Norway. Data on registrations for all refugees from Ukraine in Norway show that most travelled directly to Norway, with approximately 8.5% of registrations during 2022 reporting stays in other countries prior to arrival [26].

It is essential that areas receiving refugees are able to provide adequate and accessible health services. The NIPH report [11] found that 86% of respondents reported having needed health care in Norway. Of these, only 32% reported having received the health care they felt they needed. This was more prevalent among the more recently arrived. Barriers to access or inadequate information about the Norwegian healthcare system can impair the refugees’ health. Additionally, several social determinants can influence their health, such as income, employment, housing, and social inclusion. Ensuring that refugees receive the health care they need is essential to upholding the principles of health equity.

## Study limitations

Recruitment was self-selective, and the survey was only available digitally, such that participants may not be representative of the target group as a whole (adult refugees from Ukraine in Norway). However, the proportion of respondents arriving at different times throughout 2022 reflects the patterns of registered arrivals, with most arriving in March and April [11], as do the trends in demographics [26]. As in several other studies, highly educated women were highly represented among respondents [15, 16, 18]. Compared to official registrations in Norway, the study sample had a higher proportion of females, and the older age groups were less represented [11]. A large proportion had higher education, probably higher than the target population, however systematic information about education-level among registered refugees is not available. Higher education is known to be associated with better health, and participation in health surveys [39], and may have resulted in this study overestimating the health of the refugees. Use of technology and social media, to become aware of and access the survey, may also represent a threshold of complexity better suited for more highly educated and younger people. There may also be other differences between those who chose to participate and those who were not reached or chose not to participate.

Survey items in this study were taken from existing public health surveys [11], which allows for comparison with existing surveys, but conversely are not tailored for a refugee population specifically. Furthermore, the survey was cross-sectional, so we cannot describe changes in health status of individuals over time. Findings represent the status among refugees arriving during 2022, which may change over time and may not be representative of future arrivals. We do not have information about whether or not the survey participants sought asylum or otherwise transitioned through other countries before Norway, which could have impacted their physical and mental health as well. We also do not have information of where in Ukraine respondents moved from, and this would likely impact their health status, based on disparities in health prior to the full-scale invasion. Development in the geographical spread of the invasion will likely affect future displacement patterns. Per 30 November 2023, 79% of TCP holders in Norway come from an oblast that had been or was currently strongly affected by the war [26].

## Conclusions

Our study indicates that Ukrainian refugees who arrived later in 2022 generally reported poorer long-term health, but less psychological distress than those who arrived earlier in the same year. As these people integrate into Norwegian society and access its health care system, it will be important to provide ongoing support for their health needs. Tailored information and accessible health services likely play a substantial role in refugees’ ability to receive the health care that they need. These findings may help inform short and long-term planning of health services for refugees from Ukraine, especially in areas receiving large numbers of refugees. Future studies about physical and mental health according to time of arrival, especially for a particular group or conflict, can add to our understanding of temporal effects and better set these findings in context.

## Data Availability

The datasets generated and/or analysed during the current study are not publicly available due to privacy laws. Aggregated datasets are available from the corresponding author on reasonable request.

## Abbreviations

aOR: adjusted odds ratio
DPIA: data protection impact assessment
HSCL-5: short version of Hopkins Symptom Checklist
NCPHS: Norwegian County Public Health Surveys
NIPH: Norwegian Institute of Public Health
PTSD: post-traumatic stress disorder
T1: time period February-April 2022
T2: time period May-August 2022
T3: time period September-December 2022
TCP: temporary collective protection
TSD: Services for Sensitive Data (Tjenester for Sensitive Data)
